# Examining Levels of Risk Behaviors among Black Men Who Have Sex with Men (MSM) and the Association with HIV Acquisition

**DOI:** 10.1371/journal.pone.0118281

**Published:** 2015-02-17

**Authors:** Risha Irvin, Snigdha Vallabhaneni, Hyman Scott, John K. Williams, Leo Wilton, Xin Li, Susan Buchbinder, on behalf of HPTN 061

**Affiliations:** 1 Division of Infectious Diseases, Johns Hopkins University, Baltimore, Maryland, United States of America; 2 Bridge HIV, San Francisco Department of Public Health, San Francisco, California, United States of America; 3 Center for AIDS Prevention Studies, University of California San Francisco, San Francisco, California, United States of America; 4 Department of Psychiatry and Biobehavioral Sciences, University of California Los Angeles (UCLA), Los Angeles, California, United States of America; 5 Department of Human Development, Binghamton University, Binghamton, New York, United States of America; 6 Faculty of Humanities, University of Johannesburg, Johannesburg, South Africa; 7 Vaccine and Infectious Disease Division, Fred Hutchinson Cancer Research Center, Seattle, Washington, United States of America; 8 Department of Epidemiology and Biostatistics, University of California San Francisco, San Francisco, California, United States of America; Emory University Rollins School of Public Health, UNITED STATES

## Abstract

Seroadaptation is defined as the practice of modifying sexual behavior based on one’s own HIV serostatus, the perceived HIV serostatus of sexual partners, and differences in risk of HIV transmission by sexual acts. Because this definition implies intent, we use the term “seroprotection” to describe HIV negative participants reporting condomless anal sex (CAS) either exclusively with seronegative partners, or only as the insertive partner with HIV positive or unknown serostatus partners. Little is known about seroprotection in Black men who have sex with men (MSM). We evaluated the independent association of seroprotection and HIV acquisition among the 1144 HIV-negative Black MSM enrolled in HPTN 061 using Cox models; we stratified by city of enrollment, and controlled for number of partners, age, and drug use. Behaviors reported at 0, 6, and 12 months were assigned to three mutually exclusive categories: (1) No CAS; (2) Seroprotection; and (3) CAS without seroprotection. In 2,861 six-month intervals; 28 HIV seroconversions occurred. No CAS was reported at 33.3% of visits, seroprotection at 46.6% of visits, and CAS without seroprotection at 20.1% of visits. The seroconversion rate per 100 person-years for no CAS was 0.98 (95% CI: 0.27, 2.51), compared with 2.39 (95% CI: 1.03, 4.71) and 13.33 (95% CI: 7.62, 21.66) for seroprotection and CAS without seroprotection, respectively. Compared to CAS without seroprotection, intervals without CAS were associated with an 87% reduction (aHR: 0.13, 95% CI: 0.03–0.46) in HIV acquisition and intervals with seroprotection with a 78% reduction (aHR: 0.22, 95% CI: 0.09–0.57). No CAS is the safest behavior to prevent HIV acquisition. Seroprotective behaviors significantly reduced risk, but HIV incidence was still >2/100 person-years, suggesting that additional strategies, such as pre-exposure prophylaxis, are warranted for this population.

## Introduction

Black men who have sex with men (MSM) are disproportionately affected by the HIV epidemic in the United States (US). Approximately one quarter of all new HIV infections in the US occur among Black MSM [1]. Moreover, the Centers for Disease Control and Prevention (CDC) reports a 48% increase in HIV incidence among young black MSM between 2006 and 2009 [2]. Due to the disproportionate impact of HIV on Black MSM, HIV prevention has become a key focus area in addressing HIV-related health disparities among this group.

Seroadaptation has traditionally been defined as the practice of modifying sexual behaviors based on one’s own HIV serostatus, the perceived HIV serostatus of a sexual partner, and differences in risk of HIV transmission by sexual acts [3]. For example, seroadaptation in HIV-negative MSM can include: *serosorting* where condomless anal sex (CAS) is limited to a partner or partners believed to be HIV negative; *being an exclusive top* where all CAS is in the insertive position; and *seropositioning or strategic positioning* where CAS is in the receptive position with negative partners, but only in the insertive position with potentially serodiscordant partners. These behaviors have been widely reported among MSM and are presumably used to reduce risk of HIV acquisition and transmission [4–7]. Although seroadaptation implies intentional altering of behaviors based on partner serostatus, most longitudinal studies reporting on sexual behaviors have not asked participants about intent, but simply categorize based on reported behaviors. Therefore, the terms encompassed by seroadaptation are more accurately defined as “seroprotection” or categorizing patterns of sexual behavior (e.g., HIV negative participants reporting CAS either exclusively with seronegative partners, or only as the insertive partner with HIV positive or unknown serostatus partners), whether intentional or not. While not recommended by the CDC as an HIV prevention strategy among HIV-negative MSM, several studies have demonstrated that seroprotection, while posing a higher risk than consistent condom use, carry a lower risk of HIV acquisition than having CAS without regard to partner HIV status or sexual position [8–11]. However, many of these studies were comprised of predominantly White samples of MSM that enrolled a small proportion of MSM of color. Given the underrepresentation of Black MSM in most of these studies and the substantially higher rates of HIV infection in this population, studies focused on Black MSM are needed.

Results of previous studies examining seroprotection among Black MSM have varied. One study examining racial differences in seroprotection found that Black MSM may be less likely to report engaging in any seroprotection, and may be less likely to believe that seroprotection is an effective HIV prevention strategy [7]. The Brothers y Hermanos study of Black and Latino MSM in the US found that among Black MSM, serosorting and strategic positioning were both associated with a lower risk of HIV infection as compared to CAS regardless of partner status or sexual position [12]. However, a study of sexually transmitted disease (STD) clinic attendees from Seattle found serosorting was associated with a lower risk of HIV infection among White MSM but was not protective among Black MSM [10,13]. Further research on seroprotection among Black MSM is needed to better understand the frequency of these behaviors and how these strategies may possibly be used for harm reduction in this population.

HPTN 061 is the largest longitudinal HIV prevention study among Black MSM in the US and provides the opportunity to understand seroprotection among Black MSM. The objectives of the current analysis were to examine the prevalence and assess the effectiveness of seroprotection among Black MSM in preventing HIV acquisition.

## Methods

HPTN 061, a study designed to evaluate the feasibility and acceptability of a multi-component HIV prevention intervention among Black MSM, recruited from July 2009 to October 2010. Participants were recruited through community outreach or as sexual network partners referred by index participants in Atlanta, Boston, Los Angeles, New York City, San Francisco, and Washington DC. The institutional review boards (IRB) at all participating institutions approved the HPTN 061 study [14]. Men were eligible to participate in the study if they self-identified as a man or male at birth and as Black, African American, Caribbean Black, or multiethnic Black, were at least 18 years old, reported at least one instance of CAS with a man in the past six months, and resided in the metropolitan area and had no plans to move during the study. If men were enrolled in any other HIV interventional research study, had been a participant in an HIV vaccine trial or were a community-recruited participant in a category that had reached its enrollment cap, they were ineligible for the study. Data collection at enrollment included interviewer-collected demographic and social and sexual network information. To minimize social desirability, audio computer-assisted self-interview (ACASI) was utilized to collect self-reported data on HIV testing history and sexual risk behavior. This analysis was limited to participants who were HIV-negative, biological male, and reported behavioral characteristics during the study time period. The methods of the study have been described in detail elsewhere [14].

### Measures

In HPTN 061, behaviors reported on ACASI in the prior six months were divided into three mutually exclusive categories:

1. ***No CAS:*** intervals during which participants reported no sex of any kind, only oral sex, or consistent condom use during all anal sex episodes, regardless of partner serostatus.
2. ***Seroprotection:*** intervals in which participants reported some CAS, but that in every case during this interval, the CAS was with a partner or partners believed to be HIV negative (serosorting), was in the insertive position either with all partners (exclusive top) or only with potentially serodiscordant partners (seropositioning or strategic positioning).
3. ***CAS without seroprotection:*** all other intervals, during which participants reported some CAS without potentially protective seroprotection and engaged in receptive anal intercourse with an HIV-positive or unknown-status partner.

The above categories were adapted from previous work [5, 11]. As noted in the introduction, these sexual behaviors do not imply any intentional practice.

### Statistical Analysis

Demographic and baseline characteristics were summarized by frequency distributions (for categorical variables) or median and interquartile range (for continuous variables). Multinomial logistic regression was used to estimate the independent associations of covariates with the repeated sexual behavior measures, contrasting category 2 (Seroprotection) and category 3 (CAS without seroprotection) with category 1 (No CAS), the least risky behavior pattern. Robust standard errors were used to account for within-subjects correlation of the repeated outcomes. To assess the association between the various sexual risk categories and HIV infection, we used Cox models with the baseline hazard stratified by city. HIV acquisition was assessed at each six-month visit. Over the duration of the study, participants could be placed into a different category at each study interval, based on their reported behaviors in the previous six months. Seroprotective category was treated as a time-dependent covariate, with results summarized by relative hazards using category 1 and then category 3 as the reference level. All models were adjusted for age at enrollment, number of sexual partners and any methamphetamine, cocaine, or amyl nitrite use in the prior six months as time dependent covariates. Data analyses were implemented in Stata Version 12.0 (Stata Corp, College Station, TX) and SAS Version 9.2.

## Results

Among the 1144 Black MSM included in this analysis, 1110 completed behavioral data at baseline. The median age of the sample was 39 years. More than half of the men had a high school education or less and nearly 40% had an annual household income under $10,000 (Table 1). Cocaine/crack use was reported by 25% of men which was more common than methamphetamine (9%) or amyl nitrite (9%) use in the past six months.

**Table 1:** Demographic and behavioral characteristics of HPTN 061 participants by seroprotective behavior at baseline.

**Characteristic**	**Total N (%)**	**Category 1: No CAS N (%)**	**Category 2: Seroprotection N (%)**	**Category 3: CAS without seroprotection N (%)**
Total^1^	1110	127	654	329
**Age**				
Median	39	41	39	33
25th, 75th %tile	25, 47	27, 48	27, 47	23, 45
**Education**				
Missing	1/1110 (<1%)	0/127 (0%)	0/654 (0%)	1/329 (<1%)
Less than college	585/1110 (53%)	82/127 (65%)	342/654 (52%)	161/329 (49%)
Some college or more	524/1110 (47%)	45/127 (35%)	312/654 (48%)	167/329 (51%)
**Annual Income**				
Missing	11/1110 (1%)	0/127 (0%)	6/654 (1%)	5/329 (2%)
<$10,000	408/1110 (37%)	52/127 (41%)	227/654 (35%)	129/329 (39%)
>$10,000	691/1110 (62%)	75/127 (59%)	421/654 (64%)	195/329 (59%)
**Substance use in the past 6 months**				
Methamphetamine	101/1110 (9%)	8/127 (6%)	51/654 (8%)	42/329 (13%)
Cocaine/Crack	278/1110 (25%)	38/127 (30%)	158/654 (24%)	82/329 (25%)
Amyl Nitrite	105/1110 (9%)	5/127 (4%)	48/654 (7%)	52/329 (16%)
**Total male partners**				
Median	3	2	3	5
25th, 75th %tile	2,6	1, 3	2, 6	3, 8

^1^ Participants who are HIV negative, biological male and reported seroprotective behaviors at baseline are included in the table. Data on behavioral characteristics at baseline missing for 34 participants (total cohort N = 1144). CAS—Condomless anal sex

In multivariate modeling controlled by city, those reporting seroprotection were younger, more likely to have attended some college, and report a higher number of male partners compared to those reporting no CAS (Table 2). Those reporting CAS without seroprotection were more likely to be younger, have a higher number of partners and use methamphetamines or amyl nitrite as compared to those reporting no CAS. All 1144 participants were included in multivariate modeling as they reported behavioral characteristics at subsequent time points.

**Table 2:** Multinomial logistic regression of covariates with sexual behavior (includes all visits)^1^.

	Category 2: Seroprotection (Reference: Category 1-No CAS)			Category 3: CAS without seroprotection (Reference: Category 1-No CAS)		
Variable	RRR	95% CI	p-value	RRR	95% CI	p-value
Age (every 10 years increase)	0.87	0.80, 0.96	0.005	0.69	0.60, 0.79	<0.0001
Education (Ref: No college)	1.27	1.03, 1.55	0.02	1.17	0.87, 1.56	0.29
Income (Ref: < 10,000)	1.22	0.99, 1.51	0.06	0.94	0.70, 1.26	0.67
Number of partners (Ref: < 2 partners)	4.24	3.41, 5.26	<0.0001	11.32	8.58, 14.94	<0.0001
Substance use in the past 6 months						
Methamphetamine (Ref: No)	1.24	0.85, 1.79	0.27	1.62	1.02, 2.56	0.04
Cocaine/Crack (Ref: No)	1.05	0.81, 1.34	0.72	1.36	0.94, 1.95	0.10
Amyl Nitrite (Ref: No)	1.17	0.79, 1.74	0.43	2.00	1.26, 3.18	0.003

^1^controlled by city; CAS-condomless anal sex

Over the 12-month follow-up period, 28 of the 1144 participants HIV seroconverted (Table 3). Of 2,861 six-month intervals, 33.3% of the visits were categorized as no CAS (Category 1), 46.6% as seroprotection (Category 2), and 20.1% as CAS without seroprotection (Category 3). We built two multivariate models, each stratified by city and adjusted for age at enrollment, number of sexual partners and any methamphetamine, cocaine or amyl nitrite use. The seroconversion rate per 100 person-years for no CAS was 0.98 (95% CI: 0.27, 2.51) while the seroconversion rate for seroprotection and CAS without seroprotection was 2.39 (95% CI: 1.03, 4.71) and 13.33 (95% CI: 7.62, 21.66) respectively. Compared to CAS without seroprotection, intervals without CAS were associated with an 87% reduction (aHR: 0.13, 95% CI: 0.03–0.46) in HIV acquisition, and intervals with seroprotection with a 78% reduction (aHR: 0.22, 95% CI: 0.09–0.57). When no CAS was used as the reference category, seroprotection was not significantly associated with risk (aHR 1.78, 95% CI 0.45–6.99).

**Table 3:** Adjusted relative hazards ratio of HIV seroconversion among Black MSM in HPTN 061.

Risk category	Visits N (%)	HIV SC (N)	Seroconversion Rate (per 100 person-years) (95%CI)	Adjusted HR^1^ (Model 1) (95% CI)	Adjusted HR^1^ (Model 2) (95% CI)
Category 1-No CAS	953 (33.31%)	4	0.98 (0.27, 2.51)	Reference	0.13 (0.03–0.46)
Category 2-Seroprotection	1332 (46.56%)	8	2.39 (1.03, 4.71)	1.78 (0.45–6.99)	0.22 (0.09–0.57)
Category 3-CAS without seroprotection	576 (20.13%)	16	13.33 (7.62, 21.66)	8.03 (2.17–29.69)	Reference

^1^Adjusted for age, number of sexual partners, and any methamphetamine, cocaine and amyl nitrite use in the last six months, stratified by city. CAS-condomless anal sex; SC-Seroconversion

## Discussion

In the largest prospective cohort of Black MSM in the US, we have evaluated the independent association of progressive levels of sexual risk behaviors with HIV acquisition. In this analysis of 1144 Black HIV-negative MSM in six US cities, we found that no CAS is the behavior associated with the lowest HIV acquisition risk. Seroprotection significantly reduced HIV acquisition as compared to CAS without seroprotection, however, HIV incidence was still >2/100 person-years, a substantial rate of infection. Our data are consistent with the Brothers y Hermanos study, a cross-sectional study of Black and Latino men which separately analyzed two kinds of seroprotection: serosorting and strategic positioning [12]. In their study, the odds for testing HIV antibody-positive among Black and Latino serosorters were somewhat (but not statistically significantly) more likely to be HIV positive than men who reported no CAS, but significantly more likely to be HIV negative compared with men reporting CAS without serosorting. Their study evaluated seropositioning separately and found that it was also associated with a lower likelihood of being HIV positive as compared to receptive CAS without seropositioning [12]. In contrast, a recent cross-sectional analysis from a Seattle STD clinic found that MSM reporting only HIV negative partners were less likely to be HIV positive than MSM reporting nonconcordant CAS among White, but not Black MSM [13]. Although the Black MSM in the Seattle study had lower reported sexual risk and similar testing histories to the White MSM, they were more likely to test HIV positive which may suggest they were less likely to have accurate information about the serostatus of their partners [13]. The Seattle study results may be different from ours because of their study focusing on patients in a STD clinic, rather than participants volunteering for a longitudinal study; geographic differences (e.g. testing frequency, proportion unaware of HIV infection) between Seattle and the 6 cities represented in HPTN 061; and a cross-sectional study rather than the longitudinal analysis used in our study.

Some studies have suggested that Black MSM were less likely to report seroprotection compared to MSM from other races/ethnicities. Our study revealed that Black MSM engaged in seroprotection at proportions similar to or higher than other largely White MSM cohorts. A recent pooled analysis of North American cohorts of predominantly White MSM from vaccine and behavioral intervention studies from 1995 to 2007 with a large sample (total of 12,277 participants; 60,162 six-month intervals with 663 HIV seroconversions) found that no CAS was reported in 47.4%, seroprotection in 31.8%, and CAS without seroprotection in 20.4% of visits; our study found proportions of 33.3%, 46.6%, and 20.1% respectively [11]. Our results were also consistent with the Brothers y Hermanos study in which 42% of Black MSM reported either serosorting or strategic positioning.

Several limitations to this study exist. First, our results were obtained using self-reported behaviors with ACASI. While ACASI increases the accuracy in the reporting of sexual and substance abuse behaviors and decreases the social desirability bias, it does not eliminate bias, as evidenced by the seroconversions in those reporting no CAS. Other explanations for infections in this group are recall bias, unrecognized condom failure or HIV acquisition through other HIV risk practice such as oral sex or injection drug use. We did not have power to analyze seroprotective strategies individually, and were unable to determine which specific behaviors may have contributed to decreased risk. Finally, the median age of the Black MSM included in this cohort was 39 at baseline and so it is unclear if these findings are generalizable to young Black MSM as HIV testing frequency and proportion unaware of infection could be different from the cohort included in this study.

Because our study found high rates of Black MSM engaging in seroprotection, it will be important to understand whether Black MSM are intentionally adopting these behaviors as a harm reduction strategy, and if so, how consistent these behaviors are over time. Concerns have been expressed about promoting seroprotection for Black MSM because of the potential for high rates of undiagnosed HIV and high existing prevalence in potential partners [2,15,16]. Although seroprotection could serve as a harm reduction strategy, men in the seroprotection group had an HIV incidence of greater than 2/100 person-years, suggesting such behaviors may benefit from additional interventions, such as pre-exposure prophylaxis [17]. In addition, the effectiveness of seroprotection relies on accurate knowledge and disclosure of serostatus; unfortunately, 46% of HIV seropositive Black MSM are unaware of their status [16]. Sexually transmitted infections also increase susceptibility to HIV acquisition, and STI rates are noted to be high among Black MSM [18–20]. Through a combination of PrEP, HIV and STI testing, disclosure interventions, and possibly seroprotection, we may be able to achieve substantial reductions in HIV incidence among Black MSM.
